# Anthelmintic Baiting of Foxes against Urban Contamination with *Echinococcus multilocularis*

**DOI:** 10.3201/eid0910.030138

**Published:** 2003-10

**Authors:** Daniel Hegglin, Paul I. Ward, Peter Deplazes

**Affiliations:** * University of Zürich, Zürich, Switzerland

## Abstract

In recent years, increases in the urban fox population have been observed in many countries of the Northern Hemisphere. As a result, *Echinococcus multilocularis* has entered the urban environment. Because of a possible increased risk for alveolar echinococcosis, intervention strategies need to be evaluated. In Zürich, Switzerland, 50 praziquantel-containing baits per km^2^ were distributed monthly in six 1-km^2^ bait areas and one 6-km^2^ bait area from April 2000 through October 2001. The proportion of *E. multilocularis* coproantigen–positive fox fecal samples collected remained unchanged in six control areas but decreased significantly in the 1-km^2^ bait areas (from 38.6% to 5.5%) and in the 6-km^2^ bait area (from 66.7% to 1.8%). *E. multilocularis* prevalence in the intermediate host *Arvicola terrestris* also decreased significantly in baited areas. This controlled baiting study shows that a pronounced reduction of *E. multilocularis* egg contamination is feasible in urban areas where the organism is highly endemic.

The zoonotic tapeworm *Echinococcus multilocularis* is typically perpetuated in a wild life cycle, which includes foxes (genera *Vulpes* and *Alopex*) as definitive hosts and various rodent species as intermediate hosts (*1*). In addition, eggs are accidentally ingested by humans; the metacestodes enter mainly the liver and cause alveolar echinococcosis, a severe, sometimes fatal disease if left untreated (*2*,*3*).

Few studies have been performed on the epidemiology of alveolar echinococcosis. Risk factors for alveolar echinococcosis may include occupational and behavioral activities. However, hunters, trappers, and persons working with fur were not at increased risk for alveolar echinococcosis in South Dakota (*4*). Data from Europe have indicated that farming activities increase the risk for infection (*5*,*6*). Contamination of the rural environment with *E. multilocularis* connected with farming activities was indirectly demonstrated by high prevalences of alveolar echinococcosis in sows kept indoors but fed with grass (*7*). Areas with high water vole (*Arvicola terrestris*) densities yielded a 10-fold higher risk for human alveolar echinococcosis compared with areas with low densities of this important intermediate host (*8*). In an area where the organism is highly endemic, up to 39% of *A. terrestris* and up to 7% of dogs with free access to rodents were infected with *E. multilocularis* (*9*), and persons who have kept dogs around dwellings were at higher risk for alveolar echinococcosis on St. Lawrence Island, Alaska (*10*).

Red foxes (*Vulpes vulpes*) are likely to be the most important final host in many regions (*11*). In the past 2 decades, foxes have started to colonize in cities around the world (*12*–*14*), and evidence of the parasite cycle in urban areas is increasing (*13*,*15*,*16*). In Zürich, Switzerland, one study found that 47% of the urban fox population was infected with *E. multilocularis* (*17*).

The high number of infected foxes in cities and villages, in close contact with domestic pets and humans, could increase the risk of alveolar echinococcosis (*16*). The disease has an incubation period of 5 to 15 years; therefore, whether the actual incidence rate of alveolar echinococcosis reflects a continuing stable and low infection risk or whether the increased infection pressure in highly populated areas will lead to a delayed increase in the incidence of alveolar echinococcosis cases in the future is unclear (*3*). However, ecologic changes resulted in a very high alveolar echinococcosis prevalence of 4.0% in China, which is highly endemic for the organism (*18*). The high prevalence of *E. multilocularis* in densely populated areas and the increase of foxes living in close vicinity to humans strongly suggest that evaluating possible intervention strategies is prudent.

Few field studies focus on anthelmintic treatment of definitive hosts. Rausch et al. (*10*) demonstrated in a village that was hyperendemic for the organism (St. Lawrence Island, Alaska) that continual treatment of dogs with praziquantel reduces infection pressure of *E. multilocularis*, resulting in lower prevalence in locally trapped voles. In extended rural areas of Germany and Japan, praziquantel baits lowered the prevalence of *E. multilocularis* in foxes (*19*–*21*). These results cannot be transferred to the condition of agglomerations and urban areas, where until now no attempt has been made to evaluate an intervention strategy for foxes.

The urban cycle of *E. multilocularis* was studied intensively in Zürich, Switzerland (*16*,*17*,*22*). Analyses of fox stomachs indicated that *A. terrestris* was the most frequently consumed intermediate host (*23*), and *E. multilocularis* is highly prevalent (mean 9.1%, maximum 20.9%) in this vole species, which lives predominantly along the city border (*22*). Accordingly, the prevalence of *E. multilocularis* in foxes was significantly higher in the urban periphery than in more central areas (*17*), and the infection risk for alveolar echinococcosis might therefore be concentrated mainly in delimited areas in the urban periphery (*16*). Since urban inhabitants frequently use the zones of highest contamination for recreational activities and their domestic cats and dogs have access to infected voles, the urban periphery may represent a risk for alveolar echinococcosis.

In this controlled experimental field study, we investigated the effect of anthelmintic baiting in defined urban areas where the organism is highly endemic and tested whether *E. multilocularis* egg contamination was significantly reduced. We also examined whether, as an expected consequence, its prevalence in urban intermediate hosts diminished.

## Methods

### Study Area

The study was conducted in the community of Zürich and its surroundings. Zürich covers 92 km^2^ and has a population of 360,000. Fifty-three percent of Zürich is an urban area with industrial, commercial, and residential buildings; the other portion of Zürich is 24% forest, 17% agricultural, and 6% water. We divided this area into zones: urban, border, and periurban zone. The urban zone is mainly residential with little green space. The periurban zone consists of forests, fields, pastures, and meadows. The border zone, which divides the urban and the periurban zone, was defined as extending 250 m from the border of the urban area with buildings into the residential area of the city and 250 m into the periurban surroundings. This zone includes mostly residential areas, allotments, cemeteries, sports fields, public places, and pastures. The border zone and the periurban zone are used by the public for recreational activities.

As far as hunting is concerned, Zürich is organized as a game sanctuary and, compared to the high population density of >10 adult foxes per km^2^ (*24*), the hunting bag (foxes shot by game wardens) was relatively low during the course of this study (1.0 shot foxes per km^2^ and year).

### Baits

Commercial baits were used in the study (Impfstoffwerk Dessau Tornau GmbH, Rosslau, Germany). Each weighed 13.5 g, and the matrix consisted of Altrofox 91 (Impfstoffwerk Dessau Tornau GmbH). This matrix is the same one as in the widely used rabies vaccine bait Rabifox (Impfstoffwerk Dessau Tornau GmbH). The baits contained 50 mg of the anthelmintic praziquantel (Droncit Bayer AG, Leverkusen, Germany), a highly efficient drug against adult cestodes.

### Experiment Design

**Figure 1:**
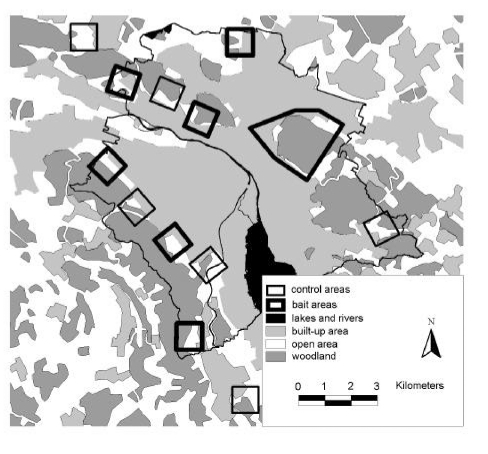
Study area of the controlled anthelmintic baiting experiment in the conurbation of the city of Zürich. 50 Praziquantel-containing baits per km^2^ were delivered monthly in six 1-km^2^ bait areas and one 6-km^2^ bait area, that alternated along the urban fringe with six control areas. Black line, Zürich border.

Along the urban periphery we selected six bait and six control areas of 1 km^2^ each and an additional bait area of 6 km^2^. Bait and control areas were separated by at least 600 m to minimize the chance of foxes using two areas (Figure 1). All areas included a similar amount of urban area with buildings, open spaces (public parks, cemeteries, allotment gardens, and meadows), and woodlands in a pattern typical for the urban fringe (Figure 1). In baited areas, 50 praziquantel-containing baits per km^2^ were distributed monthly (intervals of 25 to 35 days) during 19 months from April 2000 through October 2001. Baits were distributed manually at places that were most likely to be frequented by foxes (e.g., where fox tracks had been seen, fox dens, and compost heaps) but not by dogs. To avoid olfactory contamination, baits were always handled with rubber gloves. Baits were covered with surrounding material to protect them from sun at exposed sites.

### Sampling and Analyses of Fox Fecal Samples

Fox fecal samples were collected at least once per month in bait and control areas and their immediate vicinity during the following periods: winter 1999/2000 (November 1999 to February 2000), spring 2000 (April to June 2000), summer/autumn 2000 (July to October 2000), winter 2000/01 (November 2000 to February 2001), and summer/autumn 2001 (July to October 2001). Several criteria, such as size, shape, homogeneity, and smell of the droppings, were used to distinguish fox fecal samples from other fecal samples (*22*). For each of the 1,537 collected fecal samples, we recorded the exact position to an accuracy of 20 m.

*E. multilocularis* coproantigen was detected by a sandwich–enzyme-linked immunosorbent assay (EM–ELISA) (*25*), which was recently validated for testing field fecal samples in eastern France (*26*) and our study area (*22*). Coproantigen-positive fecal samples, collected in bait and control areas during 2001, were further evaluated to check whether infected foxes in bait areas had predominantly fresh, prepatent infections and did not excrete *E. multilocularis* eggs. Therefore, we isolated taeniid eggs from the fecal samples followed by *E. multilocularis*–specific polymerase chain reaction (PCR) as described previously (*27*).

### Sampling and Analyses of *A. terrestris*

In Zürich, we found the highest prevalence of *E. multilocularis* coproantigen in the intermediate host *A. terrestris* (*22*). Therefore, we focused on this species to evaluate the effect of bait distribution on intermediate host populations. *A. terrestris* were trapped with unbaited tong traps (Hauptner Instrumente GmbH, Dietlikon, Switzerland) and Topcat traps (TOPCAT GmbH, Wintersingen, Switzerland). Traps were set in intervals of 1 to 2 months in each bait and control area from April to November 2000 and from July to October 2001. Additionally, in the 6–km^2^ bait area, traps were regularly set from July 1999 to February 2000. All 1,229 dissected rodents were carefully examined macroscopically for lesions in their livers and other organs. Lesions >2 mm in diameter were investigated for *E. multilocularis* metacestode tissue either by examining morphologic features or by DNA detection by using modified PCR (*28*).

### Statistical Analysis

Statistical analyses were performed with SPSS–PC version 10.0. Stepwise backward logistic regression was used to test the effect of baiting on the proportion of coproantigen-positive fecal samples and on its prevalence in *A. terrestris*. The influence of baiting was represented by the interaction between the two factors: area type (baited vs. nonbaited areas) and period (temporal progress of the experiment). The area type and period variables were added as blocking variables to the initial model. In addition, season (spring: March to June, summer/autumn: July to October, winter: November to February) and urban area variables (urban zone, border zone, and periurban zone) were included in the initial model since these factors were known to affect the prevalence of *E. multilocularis* (*17*).

Deviations from expected frequencies were tested by chi square tests. P values are given two-tailed if not otherwise stated. If the minimum entry in the table of expectation was <5, p values were calculated with Actus (George F. Estabrook, New Hampshire, USA), which performs randomized contingency tables and gives probabilities for deviations from expected values (*29*). Critical significance levels were Bonferroni-corrected according to Rice, taking into account multiple tests on the same data (*30*). We calculated exact binomial 95% confidence intervals (CI) for means of binomial variables, according to the method of Clopper and Pearson (*31*).

## Results

### Baiting and Environmental Contamination

**Figure 2:**
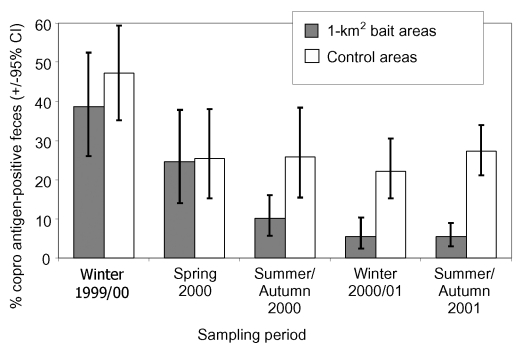
Proportions of *Echinococcus multilocularis* coproantigen–positive fox fecal samples and 95% exact binomial confidence intervals in the six 1-km^2^ bait areas, baited monthly with 50 praziquantel-containing baits per km^2^, and the six unbaited control areas during the experiment.

To evaluate the effect of the experimental baiting, we analyzed 682 fox fecal samples collected in the six 1-km^2^ bait areas and 523 fecal samples from the six control areas. The stepwise logistic regression indicated a significant final model (model χ^2^=139.4, df=11, p<0.001) with a highly significant influence of anthelmintic baiting, expressed by the interaction between the area type and period variables on the proportion of coproantigen-positive fecal samples (Wald Statistics 20.5, df=4, p<0.001). The proportion of coproantigen-positive fecal samples in bait areas decreased from 38.6% (95% CI 26.0% to 52.4%) during winter 1999 to 5.5% (95% CI 3.1% to 8.9%) in summer/autumn 2001. In the control areas, the initial proportion of coproantigen-positive fecal samples was 47.1% (95% CI 35.1% to 59.4%) in winter 1999; it decreased to 25.4% (95% CI 15.3% to 37.9%) in the initial phase of baiting (spring 2000) but thereafter remained stable during the baiting experiment (Figure 2). The two blocking factors, period (Wald Statistics=60.9, df=4, p<0.001) (Figure 2) and urban area (Wald Statistics=6.0, df=2, p=0.05), also entered the final model. In the urban zone, 1 of 33 fecal samples was coproantigen positive (mean 3.0%; 95% CI 0.0% to 15.8%), whereas within the border zone and in the periurban zone, the proportion of coproantigen-positive fecal samples was significantly higher with similar percentages of 19.1% (95% CI 16.3% to 22.0%) and 18.5% (95% CI 14.8% to 22.6%).

A strong decrease in the proportion of coproantigen-positive fecal samples was also recorded in the 332 fecal samples collected in the 6–km^2^ bait area. Before baiting started in winter 1999/2000, the proportion of coproantigen-positive fecal samples was significantly higher than in the 1-km^2^ bait areas (mean 66.7%; 95% CI 46.0% to 83.5%; χ^2^ test: p<0.05). The proportion decreased significantly to 9.2% (95% CI 3.8% to 18.1%) during summer/autumn 2000, and to 1.8% (95% CI 0.0% to 6.5%) during summer/autumn 2001 (Actus randomization test, p<0.001). This proportion of coproantigen-positive fecal samples did not differ significantly from the final proportion of positive fecal samples found in the 1-km^2^ bait areas.

**Figure 3:**
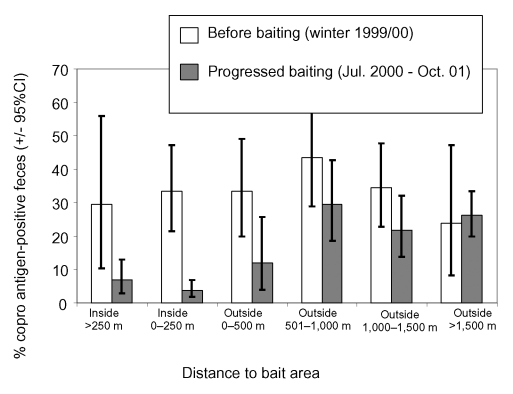
Proportions of *Echinococcus multilocularis* coproantigen–positive fox fecal samples and 95% exact binomial confidence intervals obtained at different distances from the border of the 1-km^2^ bait areas, baited monthly with 50 praziquantel-containing baits per km^2^, before baiting started (November 1999 to March 2000) and after baiting had taken place for 3 months (July 2000 to October 2001).

The spatial persistence of the baiting effect was investigated by comparing the prevalence in relation to the distance from the baiting area. In both the bait area center (>250 m inside the bait area) and in the bait area periphery (250 m inside to the border of the bait area), the effect of baiting was very pronounced (Figure 3). For fecal samples collected 0 to 500 m from the next bait area, the effect of baiting was less clear; for those collected >500 m, no significant effect could be registered.

A total of 16 coproantigen-positive fecal samples from bait areas and 55 coproantigen-positive fecal samples from control areas, all collected from July to October 2001, were investigated for the presence of *E. multilocularis* eggs. PCR analyses revealed significantly fewer fecal samples positive for *E. multilocularis* eggs (mean 25.0%; 95% CI 7.3% to 52.4%) in bait areas than in control areas (mean 52.7%; 95% CI 38.8% to 66.3%; χ^2^ test [one-tailed] p<0.05).

### Prevalence in Intermediate Hosts

**Figure 4:**
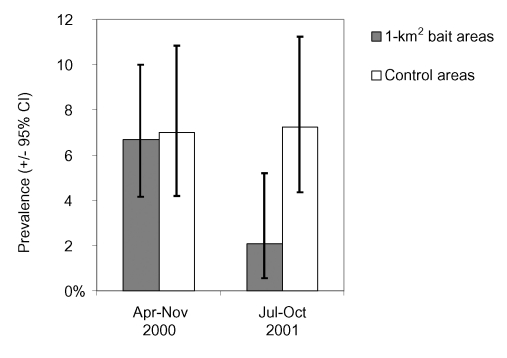
Prevalences of *Echinococcus multilocularis* in *Arvicola terrestris* and 95% exact binomial confidence intervals in the six 1-km^2^ bait areas, baited monthly with 50 praziquantel-containing baits per km^2^, and the six unbaited control areas during the experiment.

Of 1,014 *A. terrestris*, 509 originated from 1-km^2^ bait areas and 505 from control areas. The stepwise backward logistic regression indicated a significant final model (model χ^2^=8.4, df=3, p<0.05) showing that anthelmintic baiting, expressed by the interaction between area type and period, on the prevalence of *E. multilocularis* in *A. terrestris* (Wald Statistics 3.7, df 1, p [1-tailed] <0.05) had an influence. During the first year of baiting, the prevalence in control and baited areas was similar (Figure 4), but during autumn 2001 the prevalence in baited areas was significantly lower (mean 2.1%; 95% CI 0.6% to 5.2%) than in control areas (mean 7.3%; 95% CI 4.4% to 11.2%). Independently from their interaction effect, the blocking variables of period and area type also entered the final model but not urban area and season.

The results for the 1-km^2^ bait areas could be confirmed in the 6-km^2^ bait area. The prevalence of *E. multilocularis* in 215 *A. terrestris* was highest from July 1999 to February 2000 before baits were delivered (mean 21.6%; 95% CI 11.3% to 35.3%) and decreased significantly afterwards (χ^2^=4.54, df=2, p [1-tailed]=0.05). The prevalence was lower from April to November 2000 (mean 14.3%; 95% CI 6.4% to 26.2%) and lowest from July to October 2001 (mean 9.3%; 95% CI 4.5% to 16.4%).

## Discussion

### Baiting Strategy and Bait Density

The high bait density of 50 baits per km^2^, combined with a manual bait distribution at sites attractive for foxes, was highly effective. In oral rabies vaccination campaigns, up to 20 baits were usually delivered per km^2^ (*32*). Also, in the anthelmintic bait studies in Germany bait densities from 15 to 20 baits per km^2^ were successfully used (*19*,*20*). In contrast to densities in rural habitats, in urban areas, fox densities can easily exceed 10 adult foxes per km^2^ (*23*,*24*). Furthermore, in summer, many adolescent foxes are present. A previous camera trap study conducted in Zürich showed that approximately half of the baits that disappeared were taken by foxes but the others were consumed by hedgehogs, dogs, rodents, and snails (Hegglin et al., unpub. data.). Therefore, bait densities exceeding 20 baits per km^2^ seem to be appropriate to reach most foxes in urban habitats. In addition, the manual distribution of baits at selected sites attractive for foxes can improve bait uptake of foxes (Hegglin et al., unpub. data).

### Small-Scale Anthelmintic Baiting

Our results show clearly that the *E. multilocularis* egg contamination in urban areas can be reduced to a low level by manually distributing anthelmintic baits at monthly intervals. This reduction is even possible within defined urban patches of 1-km^2^ in areas where the organism is highly endemic. Although the initial rate of coproantigen-positive fecal samples was high (38.6%), this rate decreased to 5.4% during the first year of baiting. Additionally, the coproantigen-positive fecal samples in baited areas contained *E. multilocularis* eggs significantly less frequently. In contrast to our results, a large-scale praziquantel-baiting campaign in a rural area in southern Germany, covering 566 km^2^, showed a strong effect in the 156-km^2^ core area, but in the 6- to 10-km border area, the effect was much less pronounced (*19*). Immigration of young, infected foxes may have caused this border effect. In Hokkaido, Japan, an anthelmintic baiting study was carried out in a smaller, rural area of 90 km^2^, which resulted in a drastic reduction of environmental contamination comparable to our study (*21*). The main difference between the German study, the Japanese study, and our own may be explained by the different baiting strategies. In Germany, approximately half of the baits were randomly delivered by aircraft, and the intervals between two baiting actions varied from 2 to 4 months. In our study, all baits were delivered manually around places attractive for foxes at monthly intervals. A model for *E. multilocularis* control indicated that baiting intervals of 4 to 6 weeks would be most efficient (*33*).

The strong local effect in this study shows that in urban areas the population dynamics of *E. multilocularis* is mainly determined by factors of very restricted spatial extension. Knowledge about spatial dynamic of fox populations is crucial in understanding the dispersion capacity of *E. multilocularis*. Urban settings, which provide plentiful food sources, are well-suited to sustain high population densities of foxes (*34*), who tend to have small home ranges and low dispersing distances (*35*,*36*). In addition, urban fox populations are generally organized in family groups, in which predominantly young vixens remain in the parental home range and help rear pups (*36*,*37*). Consequently, offspring frequently inherit parental territory and do not have to disperse. Furthermore, a low urban immigration rate, which has been substantiated by genetic microsatellite analyses for the Zürich urban fox population (*38*), and a low hunting pressure (see Methods) contribute to the moderate spatial dynamics of the urban fox population, which we assume to be a precondition for the effectiveness of the small-scale anthelmintic treatment.

### Reduction of Infection Pressure

During the first year of baiting, when the proportion of *E. multilocularis*–coproantigen-positive fecal samples had already decreased significantly, no difference was detected in the *A. terrestris* prevalence of bait and control areas. The significantly lower prevalence of *A. terrestris* trapped in bait areas during the second year of baiting demonstrates that lower prevalence of *E. multilocularis* egg contamination resulted in a lower infection pressure for intermediate hosts. Nevertheless, at the end of the baiting study, *E. multilocularis* egg–containing fecal samples and infected intermediate host could still be detected in the 1-km^2^ and the 6-km^2^ bait areas. This finding shows that the life cycle of the parasite in the baited areas was not completely interrupted. Dispersing and transient foxes can always contaminate baited areas, even in much larger areas. Furthermore, eggs of this cestode are stable under suitable environmental conditions (*39*), infected intermediate hosts can stay infectious over several months (*40*), and baited foxes can become reinfected just after treatment by consuming an infected intermediate host. In addition, the intervention studies in Germany (*19*,*20*) demonstrated that *E. multilocularis* has the potential to recover from a population breakdown in >2 years (T. Romig, pers. comm.; K. Tackmann, pers. comm). Therefore, a baiting strategy that focuses on extinction of the parasite in large areas might fail, and permanent intervention to lower *E. multilocularis* egg contamination in defined risk areas might be more realistic and cost efficient.

## Conclusion

We demonstrated the feasibility of small-scale anthelmintic baiting of foxes to reduce *E. multilocularis* egg contamination in urban areas intensively used by the public for recreational activities, such as gardening or outdoor sports. In addition, the lower prevalence of infected voles also reduces the risk of domestic carnivores becoming infected by preying on voles and, consequently, the risk for egg transmission to pet animals. Therefore, we recommend that public health policy should focus on such defined areas where the organism is highly endemic to reduce a potential risk for alveolar echinococcosis.
